# Fungal Infections Caused by *Kazachstania* spp., Strasbourg, France, 2007–2020

**DOI:** 10.3201/eid2801.211543

**Published:** 2022-01

**Authors:** Charlotte Kaeuffer, Mathieu Baldacini, Tiffany Ruge, Yvon Ruch, Yves-Jean Zhu, Manon De Cian, Guillaume Philouze, Philippe Bachellier, Julie Denis, Nicolas Lefebvre, Francis Schneider, Yves Hansmann, Valérie Letscher-Bru, Raoul Herbrecht, Marcela Sabou, François Danion

**Affiliations:** Hôpitaux Universitaires de Strasbourg, Strasbourg, France (C. Kaeuffer, M. Baldacini, T. Ruge, Y. Ruch, Y.-J. Zhu, M. De Cian, G. Philouze, P. Bachellier, J. Denis, N. Lefebvre, F. Schneider, Y. Hansmann, V. Letscher-Bru, R. Herbrecht, M. Sabou, F. Danion);; Institut de Cancérologie Strasbourg Europe, Strasbourg (R. Herbrecht);; INSERM UMR-S1109, Strasbourg (F. Danion)

**Keywords:** fungi, Kazachstania, Candida, yeast, invasive fungal disease, emerging infection, France

## Abstract

Rare fungal pathogens are emerging as agents of invasive fungal infections. We analyzed 13 cases of fungal infections caused by *Kazachstania* (*Arxiozyma*) spp. in Strasbourg University Hospital, Strasbourg, France. Among the cases, 4 patients had proven fungal disease (3 cases of invasive fungal disease and 1 mucocutaneous infection) and 9 were colonized by *Kazachstania* (*Arxiozyma*) spp. *Candida albicans* was also isolated from 11 of the 13 patients. None of the patients with proven invasive fungal disease met host criteria, but most had underlying diseases. All strains were identified as *K. telluris* by matrix-assisted laser desorption/ionization time-of-flight mass spectrometry, and 3 were confirmed as *K. bovina* by internal transcribed spacer sequencing. For all tested strains, the MICs for fluconazole were >2 μg/mL. Emergence of this rare fungal infection might be explained by the increasing number of patients with immunocompromised conditions and gastroesophageal diseases.

## Abstract 2

Emergence might be associated with increasing immunocompromised conditions and gastroesophageal diseases.

Incidence of invasive fungal infections (IFI) has increased over the past 2 decades, mostly associated with candidemia (*1*). Rare fungal pathogens have also emerged as agents causing IFI, notably in immunocompromised persons (*2*).

*Kazachstania* (*Arxiozyma*) spp. are ubiquitous yeasts belonging to the *Saccharomycetaceae* family. *Kazachstania bovina* was described as *Saccharomyces tellustris* in 1957, as *Candida bovina* in 1958, as *Torulopsis bovina* in 1970, and finally as *K. bovina* in 2005 on the basis of multigene phylogenetic analyses (*3*–*5*). *K. bovina* belongs to the *K. telluris* species complex, which also includes *K. pintolopesii*, *K. sloofiae*, *K. heterogenica*, and *K. telluris* (*5*). Recently, a case of IFI caused by *C. bovina* (the former name of *K. bovina* in humans) was described (*6*). We report a case series of fungal infections caused by *Kazachstania* (*Arxiozyma*) spp. and classify them as invasive infections, mucocutaneous infections, or colonizations. We also describe the antifungal susceptibility testing and the methods used to identify the species.

This analysis is part of a study of opportunistic infections approved by the institutional ethics committee of the Hôpitaux Universitatires de Strasbourg. According to regulations in France, the database was declared to the Commission Nationale de l’Informatique et des Libertés. The study was registered at ClinicalTrials.gov (no. NCT03920735).

## Methods

To conduct a retrospective observational study of *Kazachstania* spp. infections, we identified all patients in the Strasbourg University Hospital, a 2000-bed tertiary-care hospital, who had *Kazachstania* (*Arxiozyma*) spp.­–positive samples during 2007–2020. We collected data on demographics, underlying diseases, clinical and radiologic aspects, mycologic results, treatments, and outcomes.

We classified *Kazachstania* spp. diseases as proven IFI according to the European Organization for Research and Treatment of Cancer and the Mycoses Study Group Education and Research Consortium updated consensus or as mucocutaneous infections (*7*). We defined colonization as isolation of *Kazachstania* spp. from a nonsterile site and absence of associated clinical and radiologic signs.

We incubated blood cultures by using Bactec Mycosis IC/F Plus Aerobic/F media (Becton Dickinson, https://www.bd.com) at 37°C. Other samples were incubated at 35°C on Sabouraud chloramphenicol agar or on chromogenic media chromID Candida (bioMérieux, https://www.biomerieux.fr) before December 2019 and CHROMagar *Candida* (Becton Dickinson) thereafter. We used a slide culture on potato carrot bile medium (Bio-Rad Laboratories, https://www.bio-rad.com) to microscopically observe *K. bovina*. 

We identified the strains by using matrix-assisted laser desorption/ionization time-of-flight (MALDI-TOF) mass spectrometry on a Microflex spectrometer and using BioTyper software (Brüker Daltonics, https://www.bruker.com). We confirmed species identification by sequencing the internal transcribed spacer (ITS) region of the ribosomal DNA with primers ITS1 and ITS4 (Eurofins Genomics GmbH, https://eurofinsgenomics.eu) (*8*). We compared sequences with those in GenBank by BLAST anlaysis (https://blast.ncbi.nlm.nih.gov/Blast.cgi) and with those in the Westerdijk Fungal Biodiversity Institute database (https://www.wi.knaw.nl). We performed antifungal susceptibility testing by using Etest or ATB Fungus 3 methods (bioMérieux). The French National Reference Center for Mycoses and Antifungals tested 1 isolate by using the microdilution method according to the European Committee on Antimicrobial Susceptibility Testing (EUCAST) guidelines (https://eucast.org) (RESSIF no. 20319).

## Results

**Figure 1:**
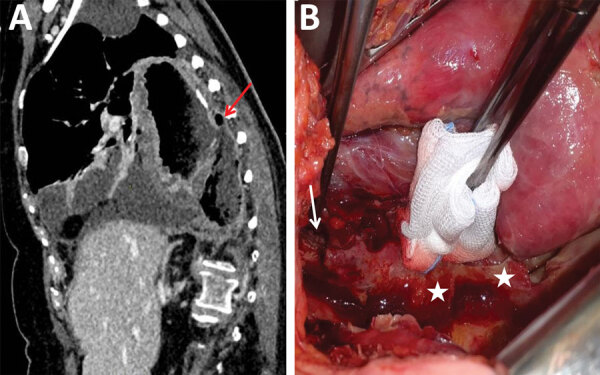
Clinical and radiologic characteristics of mediastinitis caused by *Kazachstania bovina* (patient 2), Strasbourg, France. A) Computed tomography image demonstrating stomach ulceration (arrow), mediastinitis, and pleuritis. B) Photograph taken after right-side thoracotomy, showing posterior stomach ulceration (arrow) and false membranes (stars). Culture of biopsy samples grew *K. bovina*, *Candida albicans*, *C. glabrata*, and bacteria.

We identified 13 patients with *Kazachstania* (*Arxiozyma*) spp.– positive samples. We found no temporal or spatial hospital associations between cases. Median patient age was 63 (range 40–77) years, and 7 (53.8%) patients were male (Table 1). Of the 13 patients, 4 had a proven fungal disease, of which 3 were classified as IFI: 1 case of fungemia and pyelonephritis, 1 mediastinitis (Figure 1), and 1 angiocholitis. The fourth patient had a mucocutaneous infection with biopsy-proven esophageal infection. Presence of *Kazachstania* spp. in the other 9 patients was classified as colonization. Most patients had underlying diseases that might have favored the infection or colonization, but none of the patients with proven IFI met the European Organisation for Research and Treatment of Cancer/Mycoses Study Group Education and Research Consortium host criteria (*7*). Of note, underlying esophageal pathology was reported for 4 of the 13 patients. Among the 4 patients with proven infections, treatment was caspofungin for 2 and surgery for the other 2 (in addition to proton pump inhibitor for 1 patient with esophageal infection) without any effective antifungal treatment against *Kazachstania* spp. The outcome was favorable for all 4 patients with proven infection.

**Table 1:** Clinical characteristics of *Kazachstania* spp. infections and colonizations, Strasbourg, France, 2007–2020*

Patient	Age, y/sex	Underlying condition	Exposure	Type of infection	Therapy	Outcome
1	67/F	Diabetes, endometrial cancer (remission)	Pigeon	Fungemia + UTI	FLC + CAS	Survived
2	63/F	Esophagus squamous cell carcinoma	Pigeon	Mediastinitis after gastric ulceration	CAS	Survived
3	66/M	CDP (endocrine carcinoma), recurrent angiocholitis	NA	Angiocholitis	FLC, surgery†	Survived
4	84/F	Esophageal achalasia	None	Esophagitis	PPI, surgery‡	Survived
5	68/F	CVID, gastro–jejunal anastomotic stenosis	NA	Colonization	None	Survived
6	46/M	Caustic esophageal stenosis, pneumonia	NA	Colonization	None	Survived
7	59/F	Systemic scleroderma	NA	Colonization	None	Died (cardiogenic shock)
8	40/M	Former smoker, *Staphylococcus* ventilator-associated pneumonia	NA	Colonization	None	Survived
9	51/F	AutoHSCT for oculo-cerebral NHL	NA	Colonization	FLC	Survived
10	60/M	Proven *Mycobacterium fortuitum* infection	NA	Colonization	None	Survived
11	59/M	COPD, emphysema, denutrition	NA	Colonization	None	Survived
12	77/M	Congestive heart failure, ischemic cardiomyopathy, smoker	NA	Colonization	None	Died; multiorgan failure after cardiac surgery
13	66/M	Angioimmunoblastic T-cell lymphoma, neutropenia, pulmonary tuberculosis	NA	Colonization	None	Died 5 mo later; cerebral toxoplasmosis, T-cell lymphoma progression

*AutoHSCT, autologous hematopoietic stem cell transplantation; CDP, cephalic duodenopancreatectomy; CAS, caspofungin; COPD, chronic obstructive pulmonary disease; CVID, common variable immunodeficiency; FLC, fluconazole; NHL, non-Hodgkin lymphoma; NA, not applicable; PPI, proton pump inhibitor; UTI, urinary tract infection. †Degastrogastrectomy and hepatico–jejunal and gastrointestinal anastomosis. ‡Peroral endoscopic myotomy to treat achalasia.

**Figure 2:**
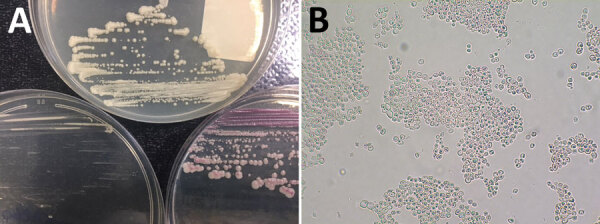
Macroscopic and microscopic examinations of *Kazachstania bovina* from a patient in Strasbourg, France. A) Macroscopic aspect of *K. bovina* on 3 agar media: Sabouraud (top), CHROMID Candida (bioMérieux, https://www.biomerieux.fr) (lower left), and CHROMagar Candida (Becton Dickinson, https://www.bd.com) (lower right). B) *K. bovina* slide-culture on potato carrot bile agar (incubation for 72 h at 27°C, original magnification ×400), showing spherical to ellipsoidal yeast cells with multilateral budding, without filamentation, and some asci containing ascospores.

The colonies appeared white on Sabouraud chloramphenicol agar and the chromID *Candida* medium and pink on the CHROMagar *Candida* medium. Growth was slower on the chromID *Candida* medium than on the other 2 media (Figure 2). A slide culture of *K. bovina* incubated for 72 h at 27°C on potato carrot bile medium showed spherical to ellipsoidal yeast cells with multilateral budding, without filamentation. Some asci containing ascospores were visible.

MALDI-TOF mass spectrometry identified all strains in this study as *K. telluris* (Table 2). Because mass spectrometry cannot distinguish between the species of the *K. telluris* complex, we identified the strains involved in IFIs, and therefore stored in our laboratory, by ITS sequencing and confirmed them as *K. bovina* (GenBank accession nos. MZ435268, MZ435269, and MZ435270). The sequences from the strains in this study were 100% similar to 2 other *K. bovina* isolates from 2 different centers (GenBank accession nos. KY103626.1 and NR_144228.1).

**Table 2:** Mycologic characteristics of *Kazachstania* spp. infections and colonizations, Strasbourg, France, 2007–2020*

Patient	Sample	Fungus species	Identification technique	Antifungal susceptibility, μg/mL						GenBank accession no.
				Method	FLC	VRC	5FC	AMB	CAS	
1	2 blood cultures, urine	*K. bovina* + *Candida albicans*	Sequencing	Etest	24	0.125	0.012	0.047	0.25	MZ435268
				EUCAST	2	<0.016	<0.125	0.015	0.015	
2	Mediastinal collection; false membranes; pleural fluid†	*K. bovina* + *C. albicans,* *+ C. glabrata*	Sequencing	Etest	8	0.125	NA	0.5	0.19	MZ435270
3	Bile (surgical sample)	*K. telluris* SC *+C. albicans*	MALDI-TOF	Etest	>256	0.19	NA	0.047	0.25	Not stored
4	Esophageal biopsy; fibroscopy: white plaques of the mucosa‡	*K. bovina* *+ C. albicans*	Sequencing	Etest	6	0.032	NA	0.125	0.25	MZ435269
5	Gastric liquid	*K. telluris* SC + *C. albicans*	MALDI-TOF		NA	NA	NA	NA	NA	Not stored
6	BAL fluid	*K. telluris* SC	MALDI-TOF		NA	NA	NA	NA	NA	Not stored
7	Stool	*K. telluris* SC + *C. lusitaniae*	MALDI-TOF		NA	NA	NA	NA	NA	Not stored
8	BAL fluid	*K. telluris* SC	MALDI-TOF		NA	NA	NA	NA	NA	Not stored
9	Urine, stool	*K. telluris* SC + *C. albicans*	MALDI-TOF		NA	NA	NA	NA	NA	Not stored
10	BAL fluid	*K. telluris* SC + *C. albicans*	MALDI-TOF		NA	NA	NA	NA	NA	Not stored
11	Sputum	*K. telluris* SC *+ C. albicans* *C. dubliniensis* *A. niger*	MALDI-TOF	AMB-fungus	4	0.25	<4	<0.5	NA	Not stored
12	Stool	*K. telluris* SC *+ C. albicans*	MALDI-TOF		NA	NA	NA	NA	NA	Not stored
13	BAL fluid, stool	*K. telluris* *+ C. kefyr*	MALDI-TOF	AMB-fungus	8	0.125	<4	<0.5	NA	Not stored

*AMB, amphotericin B; AMB; BAL, bronchoalveolar lavage; CAS, caspofungin; Etest (bioMérieux, https://www.biomerieux.fr); EUCAST, European Committee on Antimicrobial Susceptibility Testing; 5FC, flucytosine; FLC, fluconazole; ITC, itraconazole; MALDI-TOF, matrix-assisted laser desorption/ionization time-of-flight mass spectrometry; NA, not applicable; SC, species complex; VRC, voriconazole . †Anatomopathologic examination of the gastric perforation showed necrosis and inflammation. ‡Anatomopathologic examination of the esophageal biopsy showed no signs of invasion, leading to the diagnosis of mucocutaneous fungal infection.

For all strains tested, MICs for fluconazole were 2 μg/mL to >256 μg/mL (Table 2). For 11 of the 13 patients, including all with proven fungal infection, we identified another *Candida* species (most often *C. albicans*) (Table 2).

Two patients with invasive infection reported exposure to pigeons. Moreover, culture of a sample of pigeon droppings from the aviary of patient 1 enabled identification of *K. bovina* by ITS sequencing (GenBank accession no. OK037112). 

## Discussion

Among the *K. telluris* species complex, host specificity for *K. bovina* seems to be low because it has been isolated only from pigeons, a cow, and humans (*4*,*5*). To date, only 2 cases of invasive human infection caused by *K. telluris* complex have been described (*6*,*9*). One case was a *K. bovina* bloodstream infection, and the other was mediastinis caused by *K. slooffiae.* An article about extremely rare invasive fungal infections collected in the FungiScope registry did not include any cases of *Kazachstania* infection (*10*).

The isolation of *K. bovina* from 2 blood cultures from patient 1 in this study, as well as from a patient by Brunet et al. (*6*), clearly suggests pathogenicity of this fungus. Nevertheless, we identified another *Candida* species (most often *C. albicans*) in 11 of the 13 patients in our study, including all with proven fungal infection. 

Most patients had an underlying condition that might have favored the infection, including a gastroesophageal pathologic condition in 4 of the 13 patients in our study, similar to the cases reported by Brunet et al. and Mercier et al., suggesting a possible portal of entry (*6*,*9*). Moreover, 2 patients with IFI in our study reported exposure to pigeons; for 1 patient, we also isolated *K. bovina* from the pigeon droppings. Even if *K. telluris* complex in pigeons had been previously identified, to our knowledge, no cases of zoonotic transmission have been reported (*5*).

MALDI-TOF mass spectrometry identification of all strains as *K. telluris*, and further identification of 3 strains involved in IFI by ITS sequencing as *K. bovina* show the value of sequencing emerging pathogens for proper identification and epidemiology. Misidentification or incomplete identification has been noticed in a previous report of human infection with *K. bovina* (*6*).

No specific breakpoints have been established for the antifungal susceptibility of *Kazachstania* spp. EUCAST non­–species-related breakpoints for *Candida* are <2 μg/mL (susceptible) and >4 μg/mL (resistant). Our finding of fluconazole MICs between 2 μg/mL and >256 μg/mL for tested strains in our study suggests decreased susceptibility to this molecule in 5 of 7 isolates. MICs for the other antifungals tested were low for all strains. Our MIC results should be considered with caution because for 1 isolate, Etest and EUCAST indicated different fluconazole MICs. The antifungal susceptibility testing performed by the National Reference Center on Invasive Mycoses and Antifungals, using the EUCAST method on 31 strains of *Kazachstania* spp., showed high azole MICs for 55% of isolates and high caspofungin MICs for 6.5% (*11*). More recent testing of 5 *K. bovina* isolates showed fluconazole MICs to range from 2 to 8 μg/mL (*12*). To determine the susceptibility of *Kazachstania* spp. to fluconazole, the EUCAST microdilution method should be used to determine MICs for more isolates.

No specific antigens have been designed for the diagnosis of *Kazachstania* infections. We would like to have assessed the (1→3)-β-D-glucan (Fungitell, https://www.fungitell.com) panfungal antigen in the serum of the patients in our study, but this test was not available in our laboratory before 2020. However, because we identified another species of *Candida* in 11 of the 13 patients in our study, the contribution of the (1→3)-β-D-glucan test might be debatable.

The retrospective design of our study led to some limitations. We retrospectively classified cases of invasive infection or colonization, but we might have missed some information (*7*). Sequencing and antifungal susceptibility testing were not performed on all strains, notably those that were not involved in invasive fungal infections and were not stored**.**

The emergence of this very rare fungal infection might be explained by the increasing number of patients with immunocompromised conditions and gastroesophageal diseases. The use of MALDI-TOF mass spectrometry and ITS sequencing to identify yeasts might also contribute to increased documentation of these fungal infections.
